# The Benefits of Olive Oil for Skin Health: Study on the Effect of Hydroxytyrosol, Tyrosol, and Oleocanthal on Human Fibroblasts

**DOI:** 10.3390/nu15092077

**Published:** 2023-04-25

**Authors:** Anabel González-Acedo, Javier Ramos-Torrecillas, Rebeca Illescas-Montes, Víctor J. Costela-Ruiz, Concepción Ruiz, Lucía Melguizo-Rodríguez, Olga García-Martínez

**Affiliations:** 1 Biomedical Group (BIO277), Department of Nursing, Faculty of Health Sciences, University of Granada, C/Santander, 1, 52005 Melilla, Spain; anabelglez@ugr.es; 2 Biomedical Group (BIO277), Department of Nursing, Faculty of Health Sciences, University of Granada, Avda. Ilustración 60, 18016 Granada, Spain; jrt@ugr.es (J.R.-T.); rebecaim@ugr.es (R.I.-M.); crr@ugr.es (C.R.); ogm@ugr.es (O.G.-M.); 3 Institute of Biosanitary Research, ibs.Granada, C/Doctor Azpitarte 4, 4^a^ Planta, 18012 Granada, Spain; vircoss@ugr.es; 4 Biomedical Group (BIO277), Department of Nursing, Faculty of Health Sciences, University of Granada, C/Cortadura del Valle, s.n., 51001 Ceuta, Spain; 5 Institute of Neuroscience, Centro de Investigación Biomédica (CIBM), University of Granada, Parque de Tecnológico de la Salud (PTS) Avda. del Conocimiento S/N, Armilla, 18016 Granada, Spain

**Keywords:** extra virgin olive oil, fibroblasts, phenolic compounds, tissue regeneration, wound healing

## Abstract

Fibroblasts contribute to maintaining tissue integrity and homeostasis and are a key cell population in wound healing. This cell population can be stimulated by some bioactive compounds such as extra virgin olive oil (EVOO) polyphenols. The aim of this study was to determine the effects of hydroxytyrosol (htyr), tyrosol (tyr), and oleocanthal (ole) phenolic compounds present in EVOO on the proliferation, migration, cell cycle, and antigenic profile of cultured human fibroblasts. CCD-1064Sk human fibroblast cells were treated for 24 h with each polyphenol at doses ranging 10^−5^ to 10^−9^ M. Cell proliferation was evaluated using the MTT spectrophotometric technique, migration capacity by culture insert assay, and cell cycle and antigenic profile with flow cytometry. Cell proliferation was significantly increased by treatment with all compounds. The highest increases followed treatments with htyr or tyr at doses of 10^−5^ or 10^−6^ M and with ole at 10^−6^ and 10^−7^ M, and these compounds and doses were used for assays of antigenic profile, cell cycle, and migration. During the first few hours after treatment, increased fibronectin and α-actin expressions and greater cell migration were observed, with no cell cycle changes. In conclusion, these in vitro results suggest that phenolic compounds in EVOO might contribute to wound healing through action on fibroblasts related to tissue regeneration.

## 1. Introduction

The term “wound” refers to a break in the continuity of the skin and/or mucous membranes caused by physical, chemical, or mechanical agents and associated with a loss of substance or imbalanced function [1]. These events activate the dynamic and complex process of wound healing, involving hemostasis, inflammation, proliferation, and remodeling [2,3]. In the hemostasis phase, the healing process is initiated by a series of mechanisms that are set in motion to produce the hemostatic clot, thanks to the activation of the coagulation factor cascade, which results in the formation of a fibrin matrix that serves as the basis for subsequent cellular infiltration [4]. This stage is followed by the inflammation phase, which is initiated by the presence of necrotic debris, invading microorganisms, or even by the haemostatic mechanism itself, facilitating the recruitment of immune cells such as neutrophils and monocytes. This induces the liberation of pro-inflammatory mediators (e.g., TNF-α, IL-1β, IL-6, and IL-8) [5]. The proliferation phase occurs between 3 and 10 days after the wound, and is when re-epithelialisation, which consists of covering the wound surface, and angiogenesis, which aims to restore the vascularisation of the area, begins. This is accomplished by the migration of fibroblasts through fibrin networks and by the appearance of new capillaries resulting in neovascularisation [6]. Then, during the remodeling phase, the production of granulation tissue stops and the collagen fibers, that make up the damaged skin, are modified and become more resistant. Finally, as a result of this process, a mature scar is obtained which, due to the action of the myofibroblasts, decreases in size until it resembles healthy skin [7].

The role of fibroblasts in the maintenance of tissue integrity and homeostasis is crucial, as they play an essential role in the process of wound healing [8,9]. In the proliferation stage, they are responsible for breaking down the fibrin clot, and the production of collagen and elastin to form the extracellular matrix (ECM), which participates in granulation tissue formation [10,11]. The ECM plays an important role in different cellular processes, including cell adhesion, migration, the maintenance of cell shape, and proliferation [12]. However, contrary to what was initially believed, the ECM is not a fixed component of the tissue milieu that remains static and serves only as a scaffold for cells. It is well known to exert a strong influence on the cells it surrounds, including blood and lymphatic vessel cells [13]. After a wound, fibroblasts can also differentiate to become contractile myofibroblasts and proteoglycan secretors, favoring its contraction [14,15]. Myofibroblasts are defined by morphological and immunological criteria, a key aspect of their phenotype being the types of protein filaments contained in their cytoskeleton [16]. Myofibroblasts are recognized for expressing elevated levels of cytokines, ECM, and α-smooth muscle actin, which makes them crucial players in inflammation. In addition, they establish connections with components of the ECM through a transmembrane complex known as the fibronexus, which is composed of actin, integrin, and fibronectin [17].

A role in the fibroblast stimulation has been proposed for bioactive compounds in certain vegetable species, including phenolic compounds in extra virgin olive oil (EVOO). EVOO is mainly composed of triacylglycerols (~98%), fatty acids, mono- and diacylglycerols. The minor compounds of the oil represent 2% of its total weight and include more than 230 compounds, among which are hydrocarbons, sterols, aliphatic alcohols, tocopherols, pigments, volatile compounds, and phenolic compounds [18,19]. There are over 30 different types of phenolic compound present in olive oil, with hydroxytyrosol (htyr), tyrosol (tyr), and oleocanthal (ole) being present in the highest concentrations [20]. Oleo is the primary phenolic compound found in olive fruit, which can constitute up to 14% of dried fruit, while htyr is the principal phenolic component in EVOO [21]. The composition of EVOO in phenolic compounds can vary in quantity (150–700 mg/L) and quality, depending on the olive variety, degree of ripening, soil composition, climate, harvesting, processing, and storage techniques [22,23,24]. The phenolic compounds present in EVOO are renowned for their antioxidant properties, as they function as chain breakers by donating hydrogen radicals to peroxyl radicals that are produced by lipid oxidation [25]. In this respect, several studies have highlighted the antioxidant activity of htyr, tyr, and ole. Bender et al. observed a reduction in lipoxidation reactions in the blood of individuals after ingestion of a single dose of htyr-rich food supplements [26]. In the same vein, Bahrani et al. demonstrated the antioxidant effect of chitosan-lecithin-coated Parthenolide/tyr nanoparticles inhibiting ABTS and DPPH radicals [27].However, its health benefits are wider, as its regenerative, anti-inflammatory, antimicrobial potential has been demonstrated [28,29,30]. These polyphenols have also been shown to be effective in the prevention and treatment of various pathologies such as cancer, obesity, diabetes, and rheumatoid arthritis, among others [31,32,33,34,35]. Similarly, some authors have also described the effects of these phytochemicals in the management of some skin diseases such as psoriasis, atopic dermatitis, or actinic keratosis [36,37,38]. Although numerous papers have been published in the past decades regarding the advantageous effects of EVOO, there is only limited scientific evidence available on the effects of polyphenols found in EVOO on tissue regeneration. Therefore, the aim of this study was to analyse the effects of htyr, tyr, and ole, phenolic compounds present in EVOO, on the proliferation, migration, antigenic profile, and cell cycle human fibroblasts in culture.

## 2. Materials and Methods

### 2.1. Chemical Products

Reference standards of htyr [3,4-Dihydroxyphenethyl alcohol], tyr [2-(4-Hydroxyphenyl) ethanol], and ole [2-(4-Hydroxyphenyl) ethyl (3S,4E)-4-formyl-3-(2-oxoethyl) hex- 4-enoate] were acquired from Sigma-Aldrich (St. Louis, MO). These compounds were dissolved in methanol and preserved at −20 °C for utilization in the solutions under study. Analytical grade solvents or HPLC (Sigma-Aldrich), and Milli-Q grade water (Millipore Corp, Bedford, MA, USA) were used.

### 2.2. Cell Culture

The human skin fibroblast cell line CCD-1064Sk was purchased from the American Type Cultures Collection (ATCC) through the Scientific Instrumentation Center of the University of Granada. The cells were maintained in Dulbecco’s Modified Eagle Medium (DMEM) supplemented with 100 IU/mL penicillin (Lab Roger SA, Barcelona, Spain), 50 μg/mL gentamicin (Braum Medical SA, Jaen, Spain), 2.5 μg/mL amphotericin B (Sigma, St Louis, MO, USA), 1% glutamine (Sigma), 2% HEPES (Sigma), and 10% fetal bovine serum (FBS) (Gibco, Paisley, UK) under standard culture conditions (37 °C, 95% humidity, and 5% CO_2_). After reaching confluency, the CCD-1064Sk human skin fibroblast cells were harvested by treatment with 0.05% trypsin and 0.02% ethylenediaminetetraacetic acid (EDTA) from Sigma. Following detachment, cells were washed and resuspended in a culture medium containing 10% FBS for further use.

### 2.3. Cell Proliferation Assay

To evaluate cell proliferation, MTT spectrophotometry (Sigma-Aldrich Chemie) was used to measure the reduction of yellow MTT (3-(4,5-dimethyl-2-thiazolyl)-2,5-diphenyl-2H-tetrazolium bromide) to an insoluble purple formazan product [1-(4,5- Dimethyl-2-thiazolyl)-3,5-diphenylformazan] by mitochondrial succinate dehydrogenase. Initially, fibroblasts were seeded at a density of 1 × 10^4^ cells/mL per well in a 96-well plate (Falcom, Becton Dickinson Labware, NJ, USA) in estrogen-free culture medium with 10% FBS and were cultured under standard conditions for 24 h. Then, the medium was replaced with DMEM containing one of the studied phenolic compounds at doses ranging from 10^−5^ M to 10^−9^ M to cover the therapeutic dosage range [39]. The control group consisted of untreated cells that were cultured under identical conditions. After 24 h, the culture medium was replaced with phenol red-free DMEM, which was supplemented with 0.5 mg/mL MTT formazan crystals over a 4 h incubation period. Then, the cells and formazan product were solubilized with Dimethyl sulfoxide (DMSO) and absorbance was measured at 570 nm using a multiwell scanning spectrophotometer (Sunrise TM, TECAN, Männedorf, Switzerland). The percentage of cell proliferation was calculated by comparing the results with the control group [40]. Concentration–response curves were plotted, and the half maximal effective concentration (EC50) values were then calculated using a sigmoidal dose–response curve equation [41]. Only those doses of the different treatments that were effective in the proliferation experiments were used for the rest of the assays.

### 2.4. Effects of Phenolic Compounds on Cell Migration

The effect of EVOO polyphenols on fibroblast migration capacity was evaluated using the culture inserts assay technique described by Cappiello et al. [42]. Briefly, a culture insert was located in each well of a 24-well culture plate (Falcom) using sterile tongs, and 70 μL of cell suspension was added at a density of 20 × 10^4^ cells/mL. After 24 h, the culture inserts were removed, and the wells were washed with phosphate buffer saline (PBS) to eliminate any remaining cells. Then, htyr and tyr at doses of 10^−5^ M and 10^−6^ M and ole at doses of 10^−6^ M and 10^−7^ M were added. The plate was kept in a CO_2_ oven under standard culture conditions, and inverted phase contrast microscopy was used to capture images at 0, 4, 8, 12, and 24 h post-treatment. Then, Motic Images Plus software (Motic, Hong Kong) was used to analyze the gaps. The percentage of in vitro wound healing was calculated using the following formula:

Wound closure (%) = (W0 − Wn)/W0 × 100%

where W0 is the initial width of the space immediately after the culture insert assay, and Wn is the width at the different measurement time points.

### 2.5. Cell Cycle Assay

Effects of the phenolic compounds on the fibroblast cell cycle were studied by flow cytometry following Manzano et al.’s protocol [43]. Cells were first cultured in 24-well plates (Falcom) at 37 °C and 5% CO_2_ for 24 h. Then, the cells were treated with the phenolic compounds at the same concentrations used in the migration assay. After 24 h, the cells were separated and washed by centrifugation. Next, 200 μL of cell suspension in PBS at 1 × 10^4^ cells/mL was mixed with 2 mL of 70% ethanol in distilled water and vigorously agitated. The mixture was left to rest for 30 min in cold, then the cells were washed again by centrifugation and suspended in 200 μL of PBS. Subsequently, 100 μL of RNase (1 mg/mL) and 100 μL of propidium iodide were added, and the cells were cultured at 37 °C for 30 min. Finally, the cells were analyzed by flow cytometry with an argon laser at 488 nm (Fast Vantage Becton Dickinson, Palo Alto, CA, USA), and the results were expressed as the percentage of cells in each phase of the cycle (G0/G1, S, and G2/M phase distribution).

### 2.6. Antigenic Profile by Flow Cytometry

Following 24 h of exposure to the selected treatments, cells were detached from the culture flask using a 0.05% trypsin and 0.02% EDTA solution, then washed and suspended in PBS at a concentration of 1 × 10^4^ cells/mL. Cells were then labeled with monoclonal antibodies (mAbs) specific for fibronectin and α-actin, as previously described by Ramos-Torrecillas et al. [44]. Cell permeabilization was achieved using the Fix and Perm kit (Caltag Laboratories Inc, Burlingame, CA, USA), followed by incubation with 10 µL of the appropriate mAb (Table 1) in 100 µL of cell suspension for 30 min at 4 °C in darkness. After washing and suspension in 1 mL of PBS, cells were analyzed by flow cytometry (Fast Vantage Becton Dickinson) with argon laser at 488 nm to determine the percentage of fluorescent cells relative to the corresponding isotype control, based on counts of 2000–3000 cells. All cultures underwent at least three independent experiments for each antigen.

### 2.7. Immunofluorescence

Fibroblasts were treated with the selected doses of the different phenolic compounds during 24 h. The cells were fixed with a 1:1 ratio of ice-cold methanol-acetone during 10 min and then washed with PBS. Afterward, the cultures were blocked with 10% FBS diluted in PBS and incubated with the mAbs fibronectin and α-actin at a dilution of 1:500 (Table 1) for 2 h. Excess antibodies were subsequently removed, and nuclear counterstaining was performed using 4′,6-Diamidino-2-phenylindole dihydrochloride (DAPI). The staining was visualized using a Leica Spectral confocal laser microscope (Leica Microsystems GmbH, Wetzlar, Germany).

### 2.8. Statistical Analysis

The statistical analyses were performed using SPSS 26.0 (IBM SPSS, Armonk, NY, USA). The results were presented as means with standard deviations, and the normality of variable distribution was assessed with the Shapiro–Wilks test, while the homogeneity of variances was evaluated using Levene’s test. One-way ANOVA was utilized for comparing the means, and multiple comparisons were conducted using Dunnett’s post hoc test. A significance level of *p* < 0.05 was considered statistically significant. The figures were generated using Graph-Pad Prism 8 software (La Jolla, CA, USA).

## 3. Results

### 3.1. Effects of Htyr, Tyr, and Ole on the Proliferation of Human Fibroblasts in Culture

**Figure 1:**
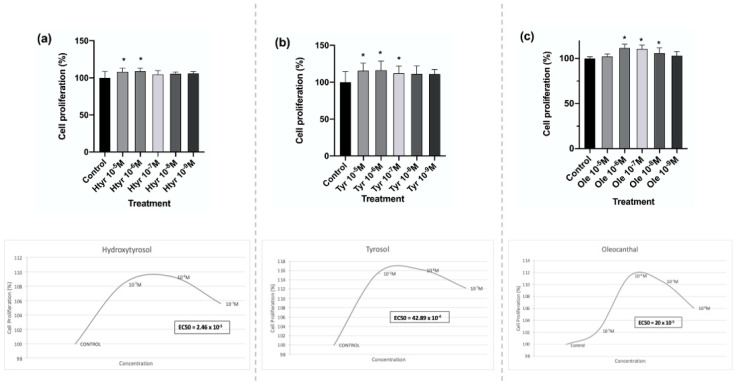
Effects of htyr (**a**), tyr (**b**), and ole (**c**) on the proliferative capacity of fibroblasts after 24 h of treatment and EC50 value for each treatment. Data are expressed in percentages with respect to results for control cells. * *p* < 0.05.

Figure 1 depicts the results of 24 h treatment with each polyphenol at the studied concentrations. In comparison with the untreated controls, fibroblasts significantly increased their growth capacity under treatment with htyr and tyr at 10^−5^ M and 10^−6^ M and with ole at doses of 10^−6^ M, 10^−7^ M, and 10^−8^ M, and most significantly by treatment with ole at 10^−6^ M and 10^−7^ M (for further information see Supplementary Materials: Table S1). The results of the half-effective concentration (EC50) are shown in Figure 1.

### 3.2. Effects of Htyr, Tyr, and Ole on the Migratory Capacity of Cultured Human Fibroblasts

**Figure 2:**
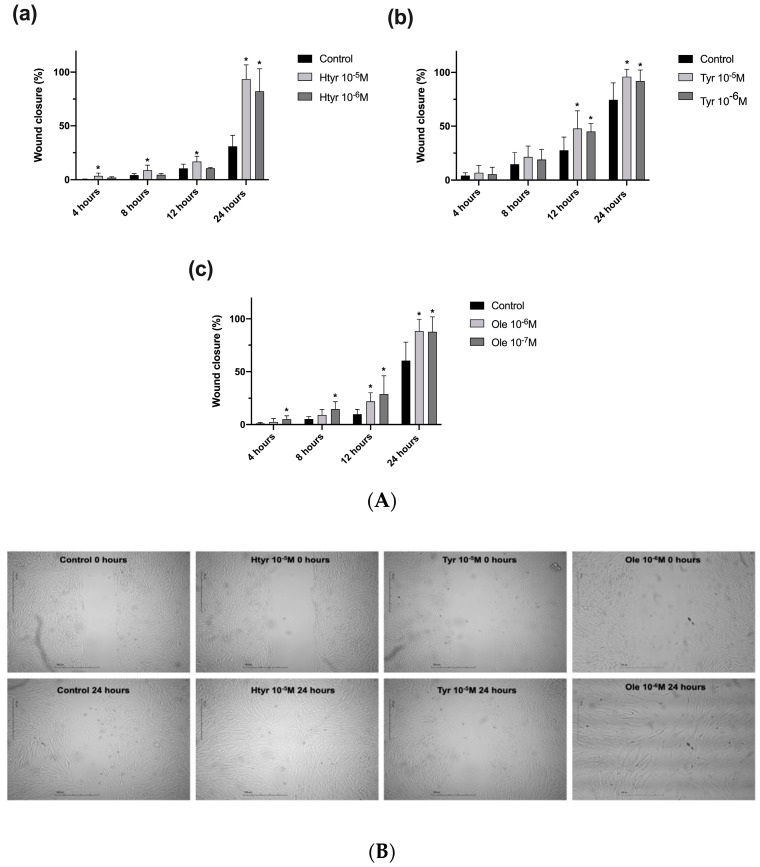
(**A**) Effect of htyr (**a**), tyr (**b**), and ole (**c**) on the migratory capacity of fibroblasts after 4, 8, 12, and 24 h of treatment. Closure of the space generated by the insert is expressed as a percentage. * *p* < 0.05. (**B**). Fibroblast migration assay: images of each treatment group after 24 h of incubation with the different polyphenols studied.

Figure 2A,B show the results of the culture insert assay on the effects of the phenolic compounds on the capacity of fibroblasts to migrate into a cell-free gap. Cells treated with htyr or tyr at 10^−5^ M or 10^−6^ M or ole at 10^−6^ M or 10^−7^ M were studied at 4, 8, 12, and 24 h (Figure 2A).

Fibroblast migration was significantly increased by htyr at 10^−5^ M from the initiation of treatment and at all time points (Figure 2A). After 4 and 8 h of treatment, this was the only compound that produced a significant increase in the percentage of closure compared with the controls. Treatment with htyr at 10^−6^ M significantly increased fibroblast migration at 24 h. The increase was highly significant (*p* = 0.0001) with both htyr doses at 24 h, coinciding with a complete closure of the cell-free space (Figure 2B). Treatment with tyr significantly increased fibroblast migration after 12 and 24 h of treatment (*p* < 0.003) at all doses (Figure 2A). Treatment with ole significantly increased fibroblast migration after 12 and 24 h of treatment (*p* < 0.002) at a concentration of 10^−6^ M, and at all measurement time points at 10^−7^ M (*p* < 0.002) (for further information see Supplementary Materials: Table S2).

### 3.3. Effects of Htyr, Tyr, and Ole on the Cell Cycle of Cultured Human Fibroblasts

**Figure 3:**
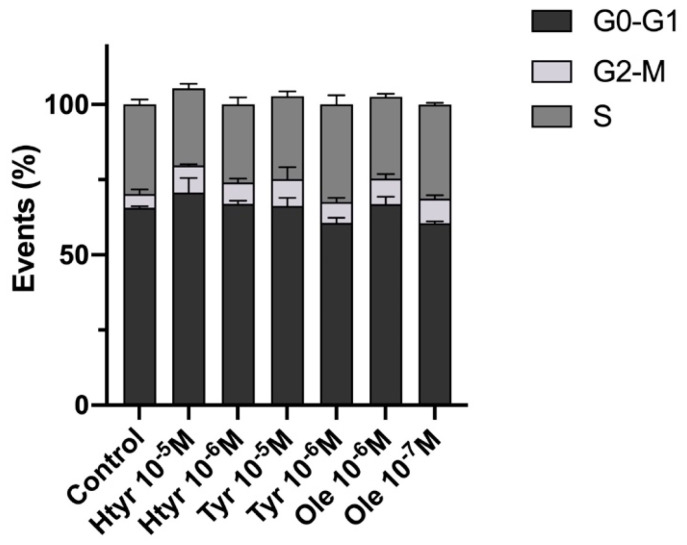
Fluorescence profile of cultured human epithelial fibroblast cell cycle at 24 h after treatment showing the percentage distribution of cells among G0/G1, S, and G2/M phases, as determined by flow cytometry.

Figure 3 depicts the cell cycle results after treatment for 24 h with htyr or tyr (10^−5^ M or 10^−6^ M) or ole (10^−6^ M or 10^−7^ M). There were no significant changes in the percentage of cells in each cell cycle phase compared with untreated cells for any of the treatments, and no signs of DNA aneuploidy or malignant transformation were detected (for further information see Supplementary Materials: Table S4).

### 3.4. Effects of Htyr, Tyr, and Ole on the Antigenic Profile of Human Fibroblasts in Culture

**Figure 4:**
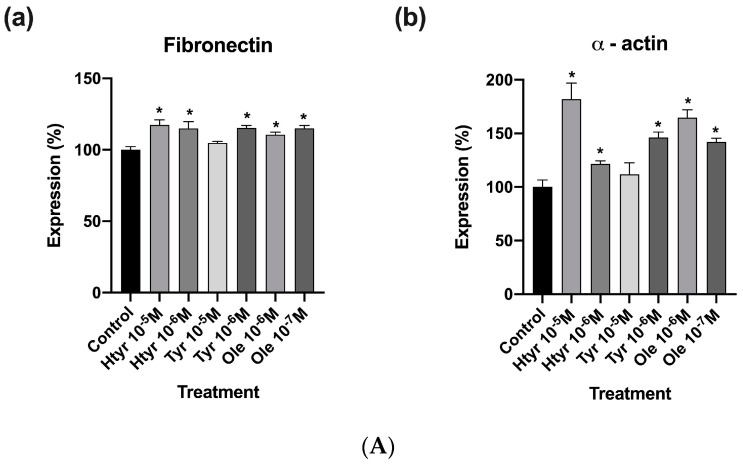

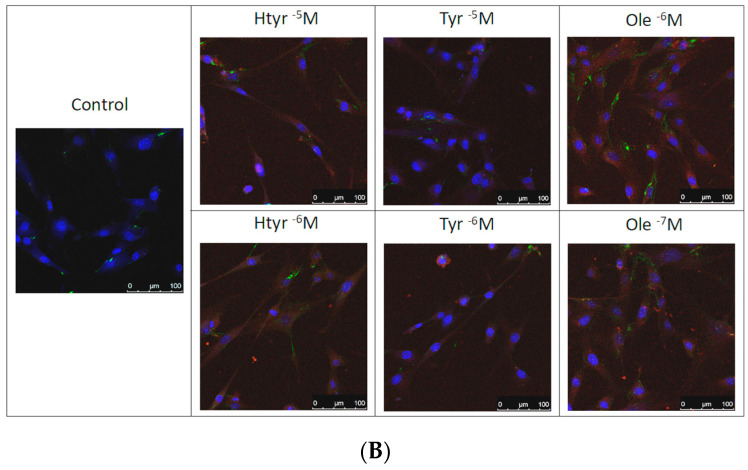
(**A**) Effect of htyr, tyr, and ole on the antigenic profile of cultured human fibroblasts. Data are presented in percentages relative to controls. * *p* < 0.05. (**a**) Effect of htyr, tyr, and ole on the expression of fibronectin in cultured human fibroblasts. (**b**) Effect of htyr, tyr, and ole on the expression of α-actin in cultured human fibroblasts. (**B**) Immunostaining with fibronectin-fluorescein and α-actin–phycoerythrin of human fibroblast cells after 24 h of treatment.

Figure 4A,B show that treatment for 24 h with htyr, tyr, and ole significantly increased the expression of fibronectin and α-actin versus controls (non-treated cells) at all the studied concentrations except for tyr at 10^-5^ M, which produced no changes with respect to untreated cells (for further information see Supplementary Materials: Table S3)

## 4. Discussion

According to this in vitro study, the phenolic compounds present in EVOO, including htyr, tyr, and ole, have been found to significantly increase the proliferation and migration of cultured human fibroblasts, enhancing their expression of fibronectin and α-actin without altering their cell cycle. These findings are consistent with those found by other authors, such as Batarfi et al. who reported similar results showing that human dermal fibroblast proliferation rates significantly increased with 0.2% and 0.4% htyr treatments compared with a control. Additionally, in the 24 h scratch assay, 0.4% htyr-treated cells exhibited increased cell migration compared with other groups, which was further confirmed in the 48 h assay [45]. Similarly, Ribeiro et al. also observed that htyr at a dose of 50 uM could promote 3T3 fibroblast cell line survival, while increasing Nrf2 expression which could promote cell migration [46].

On the other hand, our results provide insight into the processes that occur during the proliferation phase of the healing process, where there is an increase in fibroblast proliferation and migration, as observed in this study. Additionally, for effective wound healing, angiogenesis is crucial. Fibronectin has been shown to play a role in modulating the formation of new vessels by providing the necessary mechanical and chemical signals to give endothelial cells a sense of polarity during the formation of vascular tubules [47,48,49].

The proliferation stage of wound healing is also characterized by wound contraction, where myofibroblasts have an important role. These cells are believed to originate from local resident fibroblasts; according to a recent review by Hinz et al., Myofibroblasts are defined by their capability to express elevated levels of cytokines, extracellular matrix, and α-smooth muscle actin. They play crucial roles in inflammation and establish contacts with extracellular matrix components through the fibronexus [50]. From an immunohistochemical point of view, these cells exhibited positivity for several markers such as vimentin, α-actin, non-muscle myosin, extra domain A, and cellular fibronectin. Ultrastructurally, these cells are defined by the presence of prominent rough endoplasmic reticulum, a Golgi apparatus, myofilaments with focal densities, and gap junctions [51]. The observed increase in α-actin expression in our study could indicate a fibroblast differentiation process towards myofibroblasts, which would promote tissue regeneration [51,52,53,54,55].

Besides these positive effects on cell viability and wound contraction, the studied compounds may also actively participate in other processes related to wound healing, including cell adhesion, chemotaxis, and phagocytosis [56,57,58]. In this regard, several clinical trials have shown that the use of EVOO could be beneficial in the healing diabetic foot ulcers [59,60]. This could be due to its high triglyceride content, with a notable presence of oleic acid with important anti-inflammatory properties, as well as its high concentration of polyphenols with antioxidant activity [30,61,62].

Other polyphenols present in EVOO (ferulic acid, *p*–coumaric acid, caffeic acid, luteolin, and apigenin) were previously found to stimulate fibroblast proliferation and migration [63]. They also proved able to reduce the bacterial load, inhibiting the growth of multiple microorganisms, and to increase the gene expression of markers related to tissue repair (collagen I, platelet-derived growth factor, vascular endothelial growth factor, and transforming growth factor-β) [63]. The proper expression of these markers is crucial for regulating the wound healing process, and their decreased expression has been observed in chronic wounds, which may be due to a proteolytic environment [64,65,66]. Similarly, Rosillo et al. found that a polyphenol extract of EVOO, which contains htyr, ole, tyr, and other compounds such as oleuropein, apigenin, and luteolin, decreased the release of proinflammatory mediators in human synovial fibroblasts. These protective effects may be attributed to the inhibition of MAPK and NF-κB signalling pathways [67]. On the other hand, protective effects of EVOO phenolic compounds against ageing processes have been demonstrated. Thus, EVOO secoiridoids, such as oleuropein aglycon and decarboxymethyl oleuropein aglycon, have been shown to prevent age-related changes in normal diploid human fibroblasts, including alterations in cell size, morphological heterogeneity, cell arrangement, and senescence-associated β-galactosidase staining [68].

Phenolic compounds derived from a variety of vegetable species, including those commonly used in traditional medicine and present in foods such as fruits and vegetables, have been reported to have a positive impact on tissue regeneration [69,70,71,72,73,74,75,76,77]. Thus, Addis et al. found that treatment with polyphenols extracted from medicinal plants (*Calendula Arvensis*, *Lavandula stoechas*, and *Helichrysum italicum*) at a concentration of 1 μL/mL increased fibroblast cell proliferation after 24 h of treatment and stimulated cell migration after 48 h, when complete closure was observed [78]. Likewise, flavonoids from strawberries improved fibroblast cell viability, reduced intracellular reactive oxygen species, and increased mitochondrial activity, evidencing regenerative and antioxidant capacity in fibroblasts exposed to H_2_O_2_ [79]. Similar results were observed in embryonic fibroblasts co-exposed to sodium arsenite and bioactive compounds from turmeric for 24 h, which evidenced increased cell viability and reduced oxidative stress in comparison with cultures only exposed to arsenite. Then, this study suggests that polyphenols can play a protective role in the presence of toxic agents [80].

The potential of phenolic compounds in EVOO to stimulate cell growth and differentiation has been proven in other cell populations present in the skin such as HaCaT epidermal keratinocytes [81,82]. HaCaT epidermal keratinocytes exhibited an increase in proliferation after treatment with htyr at concentrations of 5 and 10 μM, likely due to the upregulation of cyclin-dependent kinases, including CDK2 and CDK6, as well as an increase in cell migration through the activation of tissue remodeling factors, such as metalloproteinase. In addition, the antioxidant properties of htyr were demonstrated by its ability to counteract the cytotoxic effects of H_2_O_2_ exposure via the activation of serine/threonine kinase, checkpoint kinase 1, checkpoint kinase 2, and p53 [81]. In the same vein, Aparicio-Soto et al. demonstrated that htyr and htyr acetate can control the inflammatory responses of human keratinocytes by inhibiting the NF-κB pathway [83]. Similar results have been obtained with compounds present in other vegetable species, e.g., resveratrol, which prevents the death of keratinocytes in a reconstructed skin model exposed to oxidative stress [84]. Fasano et al. showed that the combination of different natural compounds (coenzyme Q10, krill oil, lipoic acid, resveratrol, grape seed oil, α-tocopherol, and selenium) could attenuate oxidative stress and inflammation in human keratinocytes [85].

While this study provides encouraging findings regarding the potential use of EVOO polyphenols in promoting soft tissue regeneration, it is important to consider that the study only examined one type of cell population present in the skin+fibroblasts, while this organ is made up of other equally important cell types. Nonetheless, this research provides valuable insight into the impact of EVOO phenolic compounds on fibroblasts, and represents one of the initial studies exploring their effect on soft tissue.

## 5. Conclusions

Treatment with htyr, tyr, and ole has a beneficial effect on cultured human fibroblasts, increasing their proliferation, migratory capacity, and expression of key markers in the wound healing process without altering their cell cycle. However, further research is needed to elucidate the mechanisms of action that explain these effects, such as the antioxidant, anti-inflammatory and antibacterial properties of the compounds studied, which would contribute to the development of new therapeutic alternatives for the treatment of wounds.

## Figures and Tables

**Table 1:** Monoclonal antibodies used to mark the cells, with the fluorochrome used to label the antibody (phycoerythrin (PE) or fluoroisothiocyanate (FITC)), and the supplier.

mAbs	Fluorochromes	Supplier
Control PE	PE	Caltag (Burlingame, CA, USA)
Control FITC	FITC	Caltag
Anti-human fibronectin fluorescein	FITC	R&D Systems
Anti-human α-actin PE	PE	R&D Systems

## Data Availability

The data presented in this study are available in the supplementary material section.

## Supplementary Materials

The following supporting information can be download at: https://www.mdpi.com/article/10.3390/nu15092077/s1. Table S1: Effects of htyr, tyr and ole on the proliferative capacity of fibroblasts after 24 h of treatment. Table S2: Effects of htyr, tyr and ole on the migratory capacity of fibroblasts after 4, 8, 12, and 24 h of treatment. Table S3: Results of antigenic profile assay of human fibroblast after 24 hours of treatment with htyr, tyr and ole. Table S4: Results of cell cycle assay of human fibroblast after 24 hours of treatment with htyr, tyr and ole.
