# Supplementary material for: The Benefits of Olive Oil for Skin Health: Study on the Effect of Hydroxytyrosol, Tyrosol, and Oleocanthal on Human Fibroblasts
Source: Nutrients. 2023 Apr 25;15(9):2077. doi: 10.3390/nu15092077 (PMC10181161; doi:10.3390/nu15092077)
Supplement: Supplementary file 1 [file nutrients-15-02077-s001.zip › Supplementary Materials.docx]

**Table S1.** Effects of htyr, tyr and ole on the proliferative capacity of fibroblasts after 24 h of treatment.

| Treatment | Mean | SD | *P* |
| --- | --- | --- | --- |
| Control | 1.08 | 0.10 | - |
| Htyr 10^-5^M | 1.17 | 0.06 | 0.011* |
| Htyr 10^-6^M | 1.18 | 0.05 | 0.006* |
| Htyr 10^-7^M | 1.13 | 0.06 | 0.277 |
| Htyr 10^-8^M | 1.14 | 0.02 | 0.148 |
| Htyr 10^-9^M | 1.14 | 0.03 | 0.123 |
| Control | 0.62 | 0.09 | - |
| Tyr 10^-5^M | 0.73 | 0.06 | 0.005* |
| Tyr 10^-6^M | 0.72 | 0.08 | 0.003* |
| Tyr 10^-7^M | 0.70 | 0.06 | 0.036* |
| Tyr 10^-8^M | 0.69 | 0.07 | 0.057 |
| Tyr 10^-9^M | 0.69 | 0.04 | 0.072 |
| Control | 0.2504 | 0.0047 | - |
| Ole 10^-5^M | 0.2560 | 0.0069 | 0.408 |
| Ole 10^-6^M | 0.2792 | 0.0107 | 0.000* |
| Ole 10^-7^M | 0.2768 | 0.0108 | 0.000* |
| Ole 10^-8^M | 0.2655 | 0.0146 | 0.002* |
| Ole 10^-9^M | 0.2583 | 0.0111 | 0.168 |

* represents *p* value < 0.05 from ANOVA analysis.

**Table S2.** Effects of htyr, tyr and ole on the migratory capacity of fibroblasts after 4, 8, 12, and 24 h of treatment.

|  | 4 hours |  | 8 hours |  | 12 hours |  | 24 hours |  |
| --- | --- | --- | --- | --- | --- | --- | --- | --- |
|  | **Mean ± dt** | ***P*** | **Mean ± dt** | ***P*** | **Mean ± dt** | ***P*** | **Mean ± dt** | ***P*** |
| Control | 0.55±0.10 | ­- | 4.28±1.34 | ­- | 10.56±3.86 | ­- | 30.93±4.1 | ­- |
| Htyr 10^-5^M | 3.57±2.57 | 0.008* | 8.76±4.69 | 0.033* | 16.86±4.86 | 0.011* | 93.54±13.1 | < 0.001* |
| Htyr 10^-6^M | 1.99±0.74 | 0.283 | 4.59±1.20 | 0.979 | 10.62±0.59 | 0.974 | 82.21±2.08 | < 0.001* |
| Control | 4.17±2.71 | - | 14.77±10.77 | - | 27.66±12.21 | - | 74.60±15.75 | - |
| Tyr 10^-5^M | 6.85±6.77 | 0.422 | 21.40±10.16 | 0.190 | 47.88±16.5 | 0.001* | 94.09±6.90 | < 0.001* |
| Tyr 10^-6^M | 5.41±6.40 | 0.814 | 19.06±9.40 | 0.462 | 45.09±7.20 | 0.003* | 92.03±10.24 | 0.001* |
| Control | 1.03±0.93 | - | 5.23±2.3 | - | 9.7±4.5 | - | 60.44±17.4 | - |
| Ole 10^-6^M | 2.4±3.32 | 0.274 | 9.07±5.18 | 0.06* | 22.01±8.2 | 0.001* | 88.14±11.4 | 0.002* |
| Ole 10^-7^M | 5.02±3.13 | < 0.001* | 14.52±7.17 | 0.001* | 28.96±17.16 | 0.002* | 87.57±14.35 | 0.001* |

* represents *p* value < 0.05 from ANOVA analysis.

**Table S3.** Results of antigenic profile assay of human fibroblast after 24 hours of treatment with htyr, tyr and ole.

* represents *p* value < 0.05 from ANOVA analysis.

| Marker | Treatment | Mean | SD | *P* |
| --- | --- | --- | --- | --- |
| Fibronectin | **Control** | 75.33 | 3.46 | **-** |
|  | **Htyr 10^-5^M** | 86.27 | 2.54 | < 0.001* |
|  | **Htyr 10^-6^M** | 84.45 | 3.46 | < 0.001* |
|  | **Tyr 10^-5^M** | 76.97 | 0.83 | 0.190 |
|  | **Tyr 10^-6^M** | 84.73 | 1.22 | < 0.001* |
|  | **Ole 10^-6^M** | 81.23 | 1.25 | 0.002* |
|  | **Ole 10^-7^M** | 84.50 | 1.44 | < 0.001* |
| Actin | **Control** | 14.50 | 0.95 | **-** |
|  | **Htyr 10^-5^M** | 26.40 | 2.16 | < 0.001* |
|  | **Htyr 10^-6^M** | 17.63 | 0.40 | 0.015* |
|  | **Tyr 10^-5^M** | 16.21 | 1.56 | 0.359 |
|  | **Tyr 10^-6^M** | 21.20 | 0.72 | < 0.001* |
|  | **Ole 10^-6^M** | 23.90 | 1.05 | < 0.001* |
|  | **Ole 10^-7^M** | 20.60 | 0.52 | < 0.001* |

**Table S4.** Results of cell cycle assay of human fibroblast after 24 hours of treatment with htyr, tyr and ole.

* represents *p* value < 0.05 from ANOVA analysis.

| Phase | Treatment | Mean | SD | *P* |
| --- | --- | --- | --- | --- |
| G0-G1 | **Control** | 65.51 | 0.52 | **-** |
|  | **Htyr 10^-5^M** | 70.69 | 4.78 | 0.084 |
|  | **Htyr 10^-6^M** | 66.85 | 1.05 | 0.958 |
|  | **Tyr 10^-5^M** | 66.10 | 2.73 | 0.999 |
|  | **Tyr 10^-6^M** | 60.55 | 1.67 | 0.103 |
|  | **Ole 10^-6^M** | 66.78 | 2.46 | 0.966 |
|  | **Ole 10^-7^M** | 60.42 | 0.65 | 0.92 |
| G2-M | **Control** | 4.72 | 1.53 | **-** |
|  | **Htyr 10^-5^M** | 9.05 | 0.45 | 0.065 |
|  | **Htyr 10^-6^M** | 7.19 | 1.27 | 0.448 |
|  | **Tyr 10^-5^M** | 9.02 | 3.98 | 0.067 |
|  | **Tyr 10^-6^M** | 6.99 | 1.40 | 0.527 |
|  | **Ole 10^-6^M** | 8.57 | 1.50 | 0.112 |
|  | **Ole 10^-7^M** | 8.22 | 1.17 | 0.165 |
| S | **Control** | 29.77 | 1.64 | **-** |
|  | **Htyr 10^-5^M** | 25.65 | 4.37 | 0.066 |
|  | **Htyr 10^-6^M** | 25.97 | 2.31 | 0.097 |
|  | **Tyr 10^-5^M** | 27.59 | 1.63 | 0.524 |
|  | **Tyr 10^-6^M** | 32.46 | 3.03 | 0.328 |
|  | **Ole 10^-6^M** | 27.19 | 0.99 | 0.366 |
|  | **Ole 10^-7^M** | 31.36 | 0.55 | 0.777 |
